# LINC01134 Directly Binds and Regulates SLC1A5 Stability to Promotes Colorectal Cancer Progression

**DOI:** 10.7150/jca.100147

**Published:** 2024-10-07

**Authors:** Li Yao, Jinxiu Wu, Xiaofeng Wang, Nailing Wang

**Affiliations:** 1 Department of general surgery, Shanghai Punan Hospital, 219 Linyi Road, Pudong New Area, 200125, Shanghai, China.; 2 Department of Gastrointestinal Surgery, the First Affiliated Hospital, Sun Yat-sen University, Guangzhou 510080, Guangdong, China.; 3 Precision Medicine Institute, the First Affiliated Hospital, Sun Yat-sen University, No.1 Zhongshan 2nd Road, Yuexiu District, Guangzhou 510080, China.; 4 Department of cardiovascular medicine, Shanghai Punan Hospital, 219 Linyi Road, Pudong New Area, 200125, Shanghai, China.

**Keywords:** LINC01134, SLC1A5, RNA-RNA interaction, Colorectal cancer

## Abstract

**Background:** Colorectal cancer (CRC) is a common malignant tumor with a poor prognosis. Long noncoding RNAs (lncRNAs) have recently gained attention for their pivotal role in regulating cancer progression, including CRC. This study aimed to investigate the biological mechanisms underlying the participation of long intergenic non-protein coding RNA 1134 (LINC01134) in the progression of CRC.

**Material and Methods:** Quantitative Real-time-PCR (RT-qPCR) and western blot were applied to assess the expression levels of mRNA and protein. Functional experiments (CCK8 assay, colon formation assay, EdU assay and flow cytometry) were applied to assess cell viability and apoptosis. RNA-RNA interaction assays, subcellular fractionation analysis and dual luciferase reporter assays were employed to explore molecular interactions between LINC01134 and solute carrier family 1 member 5 (SLC1A5). The mRNA stability was analyzed using actinomycin D (ActD).

**Results:** We found that LINC01134 expression was highly expressed in CRC tissues and positively correlated with advanced clinical stages and unfavorable prognosis, which is consistent with findings from CRC cell lines. Functional experiments showed that suppressing LINC01134 restrained the proliferation of CRC both *in vitro* and *in vivo* and induced apoptosis of CRC cells. Gene co-expression analysis revealed a positive relationship between LINC01134 and SLC1A5, which was also upregulated and associated with unfavorable prognosis in CRC. Further analysis of RNA interactions and mRNA stability revealed that LINC01134 directly binds to SLC1A5 mRNA, enhancing its stability. Remarkably, silencing SLC1A5 expression partially counteracted the promotion of CRC cell proliferation by LINC01134 overexpression and alleviated its inhibition of apoptosis.

**Conclusions:** Our findings indicated that LINC01134 functioned as an oncogene in CRC by binding directly to SLC1A5 mRNA and increasing its stability. Therefore, targeting LINC01134 could be a potential therapeutic target for treating CRC.

## Introduction

Colorectal cancer (CRC) is one of the most common malignant tumors, which ranks as the second leading cause of cancer-related deaths worldwide^1^. In 2020, there were approximately 560,000 new cases of CRC and 290,000 deaths in China^2^. Due to its insidious onset and atypical early clinical symptoms, most patients with unmistakable clinical symptoms were often already in the intermediate or advanced stages of the disease^3^. The primary treatments for CRC currently consisted of curative resection combined with adjuvant chemotherapy. Despite significant advancements in new diagnostic and therapeutic approaches in recent years, the prognosis of CRC remains unfavorable, particularly in cases of metastasis^3^. One critical factor contributing to this lies in the obscure molecular mechanisms underlying the tumorigenesis and progression of CRC. Therefore, it was imperative to delve more deeply into the pivotal molecular mechanisms of CRC, which may lead to the discovery of novel biomarkers and therapeutic targets for diagnosing and treating CRC.

Long noncoding RNA (lncRNA) is defined as RNA longer than 200 nucleotides that lacks protein-coding capacity. With the rapid development of RNA sequencing and bioinformatics, an increasing number of lncRNAs had been discovered in tumors, with many studies demonstrating their involvement in various malignant biological processes such as tumor proliferation, metastasis, angiogenesis, and chemoresistance^4^. Numerous differentially expressed lncRNAs had also been identified in CRC tissues and are associated with its progression. Long intergenic non-protein coding RNA 1134 (LINC01134), a novel lncRNA, had received increasing attention in cancer research, particularly in hepatocellular carcinoma (HCC). It had been shown that LINC01134 promotes HCC progression by facilitating tumor proliferation, migration, epithelial-mesenchymal transition, oxaliplatin resistance, and radioresistance^5^-^8^. However, the potential roles and mechanisms of LINC01134 in the occurrence and development of CRC remained largely unknown.

Glutamine was the most abundant amino acid in human blood and plays a vital role in promoting cell proliferation. Solute carrier family 1 member 5 (SLC1A5), a member of the glutamine transporter family, facilitates the uptake of glutamine into cancer cells. There was accumulating evidence that SLC1A5 acted as an oncogene in various malignant tumors^9^-^11^. For instance, SLC1A5 overexpression induces glutamine-mediated ATP production, which consequently leads to gemcitabine resistance in pancreatic cancer cells^10^. Kosuke Toda and his colleagues demonstrated that knockout of the SLC1A5 gene can suppress vascular invasion and cell migration in Kras mutant CRC^12^. However, despite the critical role of SLC1A5 in cancer initiation and development, the potential regulatory mechanism of SLC1A5 involving lncRNAs remained incompletely understood.

In this study, we investigated the expression levels and prognostic role of LINC01134 in CRC. Additionally, we conducted a series of functional experiments to determine the potential impact of LINC01134 on CRC. We performed bioinformatics analyses followed by experimental validation to uncover the downstream mechanisms regulated by LINC01134. Based on our findings, we hypothesized that LINC01134 promotes CRC progression by modulating the expression of SLC1A5. Our results suggest that the LINC01134/SLC1A5 axis plays a crucial role in the development of CRC, highlighting its potential as a promising diagnostic and therapeutic target for this disease.

## Materials and Methods

### Clinical samples and cell lines

We collected 60 pairs of CRC tissues and their corresponding adjacent normal tissues from patients who underwent surgery at Shanghai Punan Hospital of Pudong New District between January 2020 and May 2021. Patients included in this study did not receive any chemotherapy, radiotherapy, immunotherapy, or other neoadjuvant therapies prior to surgery. The Ethics Review Committee of Shanghai Punan Hospital approved this study, and all participants provided written informed consent. The study was performed in accordance with the principles of Declaration of Helsinki.

We obtained Human Colonic Epithelial Cells (HCoEpic), as well as CRC cell lines (HCT116, LoVo, DLD-1, SW480, and HT-29) and 293T cell lines from the American Type Culture Collection (ATCC, Manassas, VA). All cell lines were cultured in DMEM or 1640 medium (Gibco, Waltham, MA) supplemented with 10% fetal bovine serum and incubated at 37°C with 5% CO2 using a Thermo incubator.

### Bioinformatics analysis

We analyzed gene expression levels using the online tool GEPIA (http://gepia.cancer-pku.cn/)^13^ , which integrates data from The Cancer Genome Atlas (TCGA) and the Genotype Tissue Expression (GTEx) portal. For survival analysis of the TCGA CRC dataset (TCGA-READ and TCGA-COAD), we used both GEPIA and R software (https://www.r-project.org/) with the "stat" package for co-expression analysis of LINC01134 and differentially expressed genes (DEGs), as determined by the TCGA-COAD and TCGA-READ datasets. Additionally, we performed correlation analysis using the StarBase database v2.0 (https://starbase.sysu.edu.cn/index.php/)^14^.

### Quantitative real-time polymerase chain reaction (qRT-PCR)

The study employed quantitative real-time PCR (qRT-PCR) to determine the expression levels of LINC01134 and SLC1A5. Total RNA from both tissues and cell lines was isolated using RNAzol® RT as per the manufacturer's instructions provided by GeneCopoeia, Rockville, MD, USA. Complementary DNA (cDNA) was synthesized from the total RNA through reverse transcription using a kit obtained from Thermo, USA. The expression levels were determined using a SYBR GREEN kit (TaKaRa, Kusatsu, Japan), and Roche LightCycler480® Probe Master reagent (Roche, Basel, Switzerland) was used for detection. GAPDH and U6 were utilized as internal reference controls, and the 2^-ΔΔCt^ method was applied to calculate the relative expression of the target genes to the internal references. Supplementary Table S1 lists all the primer sequences used.

### Lentiviral construction and cell transfection

To inhibit LINC01134 expression, RiboBio (Guangzhou, China) synthesized two independent synthetic cDNA oligonucleotides (sh1-LINC01134 and sh2-LINC01134) along with a negative control. These were then cloned into the expression pRNAT-U6.1/Neo vector. The full-length sequences of LINC01134 were generated by PCR and subcloned into the pcDNATM3.1(+) vector (Invitrogen) to construct the LINC01134 overexpression vector. Lentiviral expression vectors carrying sh-LINC01134, shNC, oe-LINC01134, and an empty vector were also constructed by co-transfecting recombinant and lentiviral packaging plasmids into 293T cell lines using Lentiviral Packaging Kit following the manufacturer's instructions from RiboBio (Guangzhou, China). Cell culture supernatant was collected at 48h and 72h post co-transfection and passed through 0.45 µm-pore size filters (Miltenyi Biotec). The lentivirus was harvested by centrifugation at 50000 ×g at 4°C for 90 minutes. Small interfering RNA (siRNA) of SLC1A5 and negative control RNA were obtained from RiboBio (Guangzhou, China), and their sequences are listed in Supplementary Table S2.

Stable cell lines with LINC01134 knockdown or overexpression were established by infecting them with lentivirus containing sh1-LINC01134, sh2-LINC01134, shNC, oe-LINC01134, or an empty vector in the presence of 8ug/ml polybrene. At 72h after infection, cells were selected with 5 μg/ml puromycin (Sigma) for 2 weeks. The transfection of si-SLC1A5 was performed using Lipofectamine 3000 (Invitrogen) as per the manufacturer's instructions.

### Cell proliferation assays

Cell viability was assessed using Cell Counting Kit-8 (CCK-8) (KeyGen Biotech, Nanjing, China), clone formation assays, and 5-ethynyl-2'-deoxyuridine (EdU) (RiboBio, Guangzhou, China) assays following the manufacturer's instructions. For CCK-8 assays, 1000-3000 logarithmically growing CRC cells were seeded in 96-well plates and incubated with CCK-8 solution (10 µl/ml) for 2h. The optimal density (OD) value was then measured at 450 nm on a microplate reader, and the cell viability was assessed daily for 5 days. The proliferation ability of CRC cells was evaluated through clone formation assays. Logarithmically growing CRC cells were plated in 6-well plates at a density of 1000 cells per well and cultured for 2-3 weeks. The cells were then stained with crystal violet after fixation by 4% paraformaldehyde, and the rate of colony formation was calculated from the number of seeded cells and colonies. EdU assays were conducted by seeding logarithmically growing CRC cells in 96-well plates and incubating them with 50 μM EdU reagent for 2h to incorporate EdU into DNA during replication. The cell nuclei were then stained with DAPI for 20 min and observed using a fluorescence microscope (Olympus, Tokyo, Japan).

### Flow cytometry analysis of cell apoptosis

Cell apoptosis was analyzed through flow cytometry using an Annexin V-APC/PI Apoptosis Detection Kit (KeyGen Biotech, Nanjing, China). Transfected cells were collected and resuspended in 500 µl binding buffer. Then, 5 µl Annexin V-APC and 5 µl PI were added sequentially to the cells and incubated for 15 min at room temperature away from light following the manufacturer's instructions. The results were acquired via flow cytometry (BD Biosciences) and analyzed using FlowJo V10 software.

### Western blot

Protein expression levels were analyzed by Western blotting. Total protein was extracted from clinical samples or cell lines using RIPA lysis buffer with 1% PMSF protease inhibitor. Protein concentration was determined using a BCA protein quantitation kit. Proteins were separated via 10-12% SDS-PAGE and transferred to a PVDF membrane. The membrane was blocked with 5% skimmed milk for 1 hour at room temperature, then incubated overnight at 4°C with primary antibodies (β-actin, SLC1A5). After washing, the membrane was incubated with HRP-conjugated secondary antibodies for 1 hour at room temperature. Detection was performed using an enhanced chemiluminescence (ECL) kit and visualized with the ChemiDoc Touch imaging system (Bio-Rad, Santa Rosa, CA, USA). Protein band intensities were analyzed with Image Lab software. β-aactin served as internal controls. Antibody details are provided in Supplementary Table S3.

### Mouse xenograft models

In order to explore the biological functions of LINC01134 *in vivo*, subcutaneous xenograft nude mouse models of human CRC were established using stable LINC01134-overexpression HCT116 cell lines and negative control cells. Male BALB/c nude mice aged 4-6 weeks were obtained from the animal center of Nanjing University. A density of 10 million cells/mL was mixed with matrigel and seeded at a rate of 2 million cells per 200 μl via subcutaneous injection. Subcutaneous tumors were measured every 5 days, and the mice were sacrificed 25 days after implantation. This experiment was conducted according to the National Institutes of Health Guide for the Care and Use of Laboratory Animals and approved by the Institutional Animal Care and Use Committee of Shanghai Punan Hospital.

### Immunohistochemistry (IHC)

Immunohistochemistry (IHC) was performed on paraffin-embedded sections following standard protocols. The sections were dewaxed and rehydrated, followed by antigen retrieval using a citrate-based solution. After blocking with 10% BSA, the sections were incubated with the primary antibody: anti-Ki67 (CST, Beverly, MA, USA) overnight at 4°C. Subsequently, the sections were treated with HRP-conjugated secondary antibodies. SignalStain® DAB Substrate was then applied for chromogenic detection, and sections were counterstained with hematoxylin. Finally, the sections undergo dehydration through ethanol and xylene washes before being mounted with coverslips using a mounting medium.

### RNA-RNA interaction assays *in vitro*

The Magna RIP RNA-Binding Protein Immunoprecipitation Kit (Millipore, Billerica, MA) was used to explore RNA interactions, following the manufacturer's instructions as previously described^15^, ^16^. LINC01134 and LINC01134 antisense probes labeled with BrU were obtained from Genepharma (Shanghai, China). Protein A/G Magnetic Beads (Pierce) were incubated with 5 μg anti-BrdU (ab2284, Abcam) for 30 min at room temperature. Then, the beads were washed twice and incubated with 5 pmol of BrU-labeled RNAs (LINC01134 and LINC01134 antisense) for 2 h at 4 °C. Subsequently, RIP Immunoprecipitation Buffer, including 2.5 pmol of the SLC1A5 5'UTR, CDS, 3′UTR RNA fragment, was incubated with BrU-labeled RNA probed antibody-magnetic bead labeling overnight at 4 °C. The immunoprecipitated RNAs were purified using Trizol reagent (Invitrogen) and detected by qRT-PCR.

### Subcellular fractionation

RNA fluorescence *in situ* hybridization (FISH) was conducted to identify the subcellular localization of LINC01134 and SLC1A5. Probes labeled with cy3 at its 5' end for LINC01134 and probes labeled with FAM at its 5' end for SLC1A5 were obtained from GenePharma (ShangHai, China). The hybridization was performed on CRC cell lines following the manufacturer's instructions, and cell nuclei were counterstained with DAPI. Additionally, cytoplasmic and nuclear RNA were determined using the PARIS kit (Ambition, Life Technologies) and analyzed by qRT-PCR.

### Dual luciferase reporter assays

The 5'UTR, coding sequences (CDS), and 3′UTR of SLC1A5 were cloned into the luciferase reporter vector pmirGLO (Promega Corporation). Additionally, promoter sequences of SLC1A5 were inserted into the PGL3 vector. 293T cells were cultured and seeded into 12-well plates, then co-transfected with oe-LINC01134 plasmids and either Luc-SLC1A5-5'UTR, Luc-SLC1A5-CDS, Luc-SLC1A5-3′UTR, or pGL3-SLC1A5 using the Lipofectamine 2000 Reagent (Invitrogen) according to the manufacturer's instructions. After 48 hours, cells were lysed using the Dual-Luciferase reporter assay system kit (GeneCopoeia, Rockville, MD).

### Analysis of mRNA stability

To determine mRNA stability, the transcription inhibitor actinomycin D (ActD) was used to block new RNA synthesis. SLC1A5 mRNA expression was measured via qRT-PCR in LoVo cells stably overexpressing LINC01134 and empty controls, following treatment with 10 μg/ml ActD or control, for 0, 1h, 2h, 4h, and 6h. The half-life of SLC1A5 mRNA was determined by comparing transcript levels before and after the addition of ActD.

### Statistical analysis

All experiments were performed in triplicate, and data from three independent experiments were pooled. Gene expression data were expressed as mean ± standard deviation (SD) and analyzed using the Mann-Whitney nonparametric test. Survival analysis was conducted using the Kaplan-Meier method and analyzed with the log-rank test. Categorical data were analyzed via chi-square or Fisher's exact test. Unpaired t-tests or one-way ANOVA tests were used for all other experiments. The composition ratio between groups was compared using a t-test. Statistical analysis was performed using SPSS 22.0 software (SPSS, Chicago, IL) and Graphpad Prism version 8.0 (GraphPad Software Inc, La Jolla, CA). A p-value less than 0.05 was considered statistically significant.

## Results

### LINC01134 is upregulated in CRC and correlated with a poor prognosis of CRC

**Figure 1:**
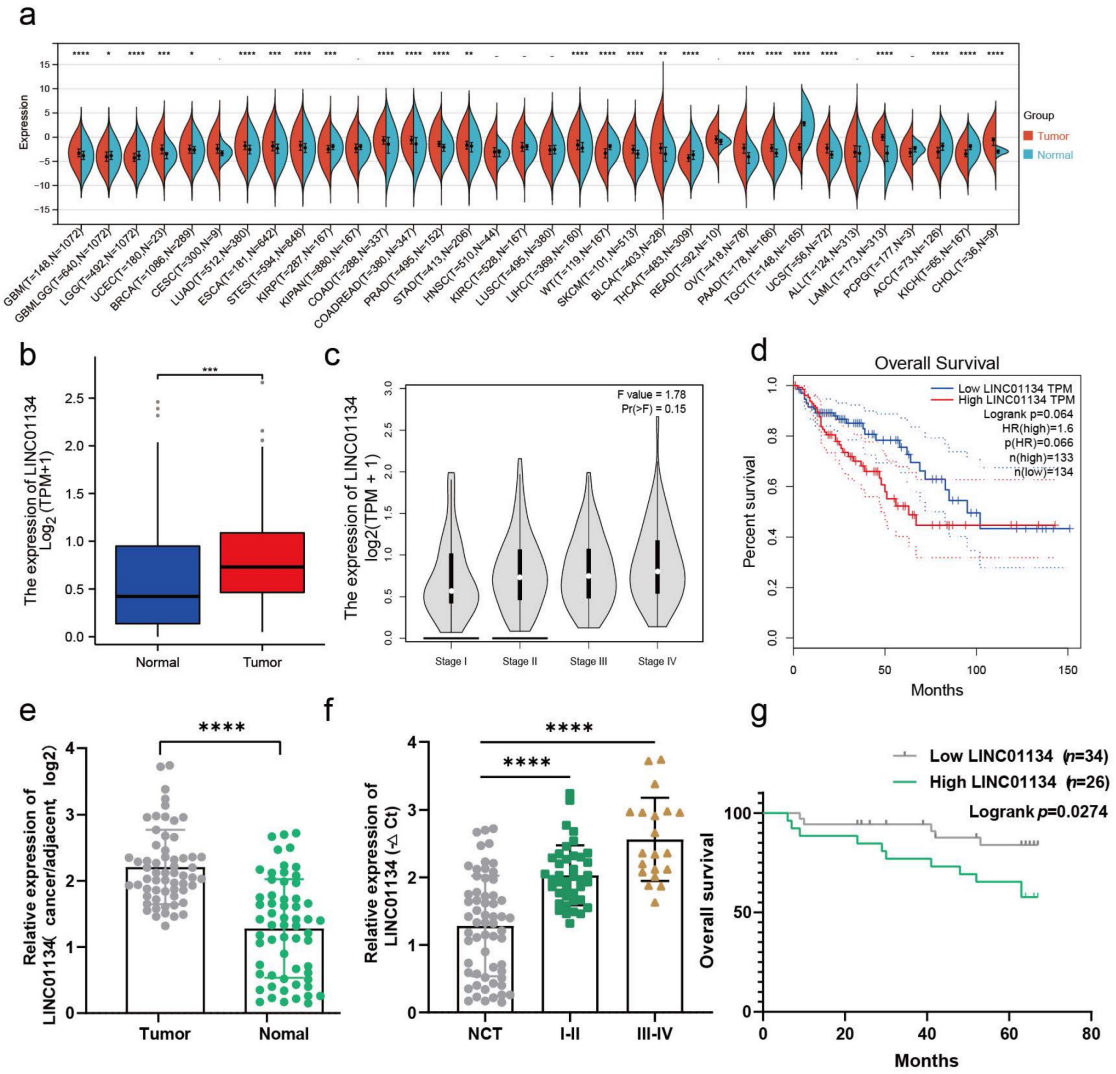
LINC01134 is upregulated and correlated with a poor prognosis in CRC. (a) The expression profile of LINC01134 in pancancer; (b-d) The expression levels and survival analysis of LINC01134 based on the Cancer Genome Atlas (TCGA) and the Genotype Tissue Expression (GTEx) portal via GEPIA and R software; (e) The expression of LINC01134 in CRC tissues (n=60) and compared normal controls (n=60) was detected by qRT-PCR; (f) The association between the expression levels of LINC01134 and clinical stages in CRC; (g) The Kaplan-Meier OS analysis of LINC01134 in CRC patients according to the expression level of LINC01134. All experiments were conducted in triplicate. ns, not significant; *p<0.05, **p<0.01, ***p<0.001, ****p<0.0001.

Previous studies have demonstrated that LINC01134 is highly expressed in CHOL, COAD, LAML, LIHC^5^, ^6^, ^17^, PRAD, and READ tissues compared to corresponding normal tissues, which was confirmed via Sangerbox 3.0 online tool (http://www.sangerbox.com/home.html) (Figure 1a). Therefore, we conducted further analyses to investigate the correlation between LINC01134 and CRC using the online tool GEPIA (http://gepia.cancer-pku.cn/detail.php)^13^ and TCGA-READ and COAD databases. The results showed that LINC01134 was significantly overexpressed in CRC tissues compared to normal colorectal tissue and correlated with advanced clinical stages (Figure 1b, c, Figure S1a). And survival analysis of the TCGA CRC dataset using R software indicated LINC01134-highly expressed CRC had shorter progression free survival (PFS) (Figure S1b), while there was no significant difference in overall survival (OS) between high and low levels of LINC01134 expression in CRC patients (Figure 1d, P=0.066, log-rank test). To verify the expression of LINC01134 in CRC tissues, quantitative RT-PCR (qRT-PCR) was conducted in 60 paired CRC tissues and adjacent normal colorectal tissues. The results showed that LINC01134 was highly expressed in CRC patients (Figure 1e), and patients with LINC01134-highly expressed CRC had worse clinical stages (Figure 1f) and shorter overall survival (OS) (Figure 1g; P=0.0274, log-rank test). Furthermore, we analyzed the correlation between the expression levels of LINC01134 and clinical characteristics, and the results indicated that histologic classes, clinical stages, and tumor metastasis were positively correlated with LINC01134 expression (Table 1).

### Silencing LINC01134 attenuated CRC cells proliferation and induced apoptosis both *in vitro* and *in vivo*

**Figure 2:**
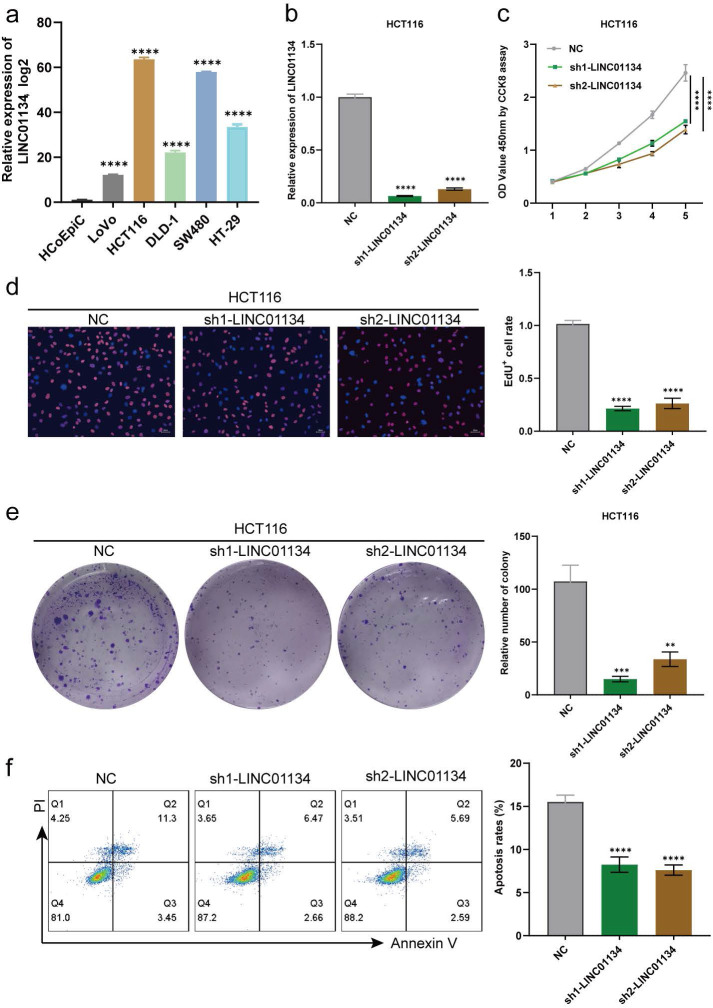
Silencing of LINC01134 attenuated CRC cell proliferation and induced apoptosis *in vitro*. (a) Expression level of LINC01134 in CRC cell lines was detected by qRT-PCR; (b) The expression level of LINC01134 in stablely LINC01134-knockdown HCT116 cell lines and negative control was assessed by qRT-PCR; The proliferation of CRC cell lines was measured by CCK8 assay (c), colon formation assay (d), and EdU staining (e); (f)The cell apoptosis of CRC cell lines was conducted by flow cytometry. All experiments were conducted in triplicate. **p<0.01, ***p<0.001, ****p<0.0001.

qRT-PCR was used to determine the expression levels of LINC01134 in CRC cell lines (HCT116, SW480, DLD-1, LoVo, and HT-29) and normal colorectal cell line (HCoEpiC), with findings indicating that LINC01134 was upregulated in CRC cell lines compared with HCoEpiC. Among the CRC cell lines, HCT116 exhibited the highest expression level of LINC01134, whereas LoVo displayed the lowest (Figure 2a). The upregulation of LINC01134 in CRC tissues and cell lines prompted us to explore its biological effects on CRC progression. We constructed stable LINC01134-knockdown HCT116 cell lines using two lentiviruses that loaded specific shRNAs, and confirmed knockdown efficiency via qRT-PCR (Figure 2b). The cell counting kit-8 (CCK-8) assay, colon formation assays, and 5-ethynyl-2'-deoxyuridine (EdU) assay were performed to measure cell proliferation. Our results showed that the proliferation of CRC cell lines was suppressed in the LINC01134-knockdown group compared with the negative control, as indicated by CCK-8 and EdU assays (Figure 2c, d). Additionally, colon formation assays revealed that downregulated LINC01134 significantly inhibited the colony formation of CRC cell lines (Figure 2e). Flow cytometry analysis was used to examine CRC cell apoptosis, and these findings indicated that knockdown of LINC01134 promoted apoptosis of CRC cells (Figure 2f).

**Figure 3:**
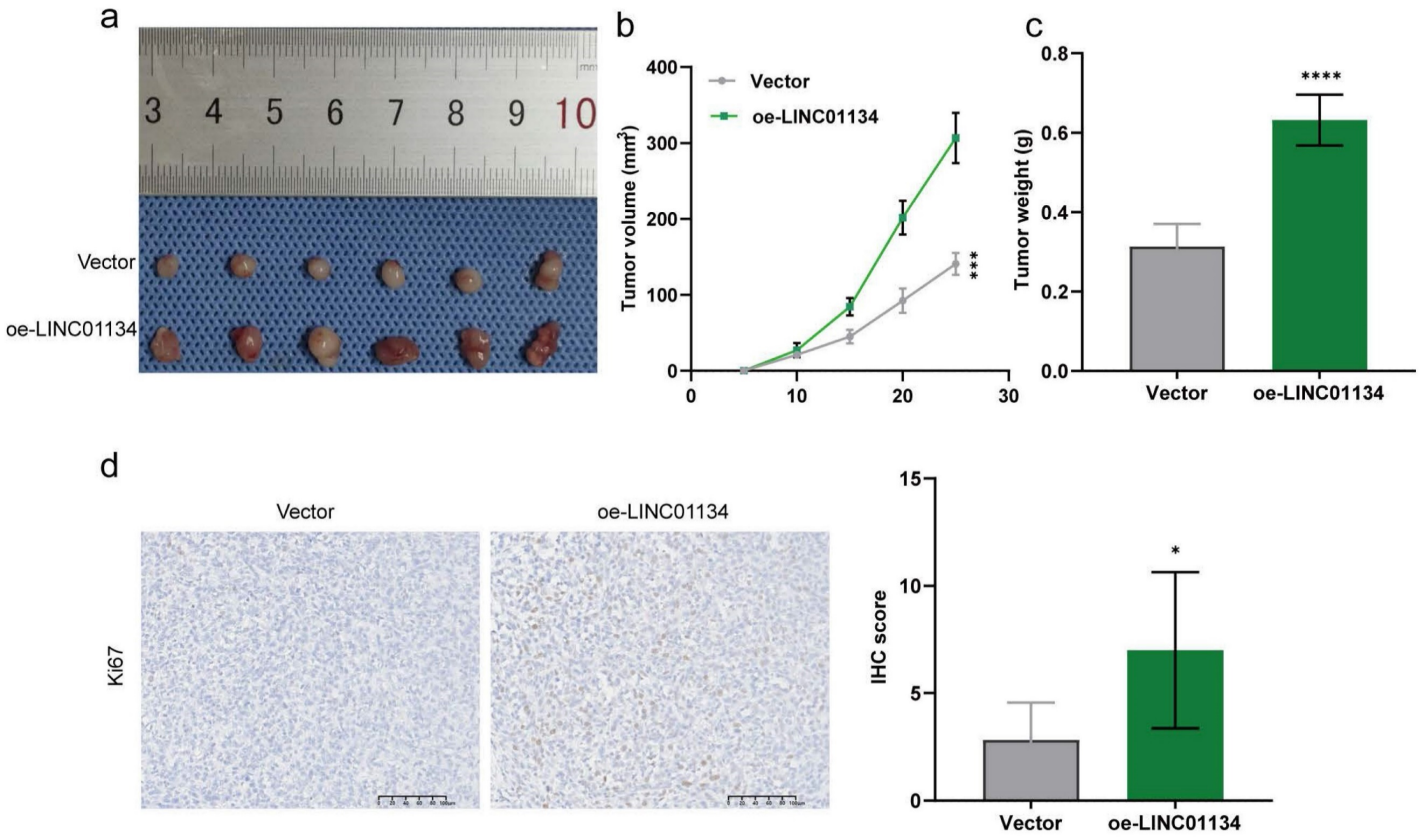
Upregulation of LINC01134 promoted CRC progression *in vivo*. Subcutaneous xenografted nude mouse models of human CRC were established to assess the effect of LINC01134 on CRC(a), the tumor volume (b) and tumor weight (c) were measured; (d) Representative images of Ki67 staining of subcutaneous tumor tissues. *p<0.05, ****p<0.0001.

To further verify the effect of LINC01134 on CRC progression *in vivo*, we established subcutaneous xenografted nude mouse models of human CRC using stable LINC01134-overexpressing HCT116 cell lines. Our results showed that overexpression of LINC01134 promoted the volume of subcutaneous xenograft tumors compared to vector control (Figure 3a-c, Figure S2). Additionally, IHC staining confirmed that high LINC01134 expression was associated with increased Ki67 staining (Figure 3d). Collectively, our findings suggest that silencing of LINC01134 attenuates CRC cell proliferation and induces apoptosis both *in vitro* and *in vivo*.

### A positive correlation between the expression of SLC1A5 and LINC01134 in both CRC tissues and cell lines

**Figure 4:**
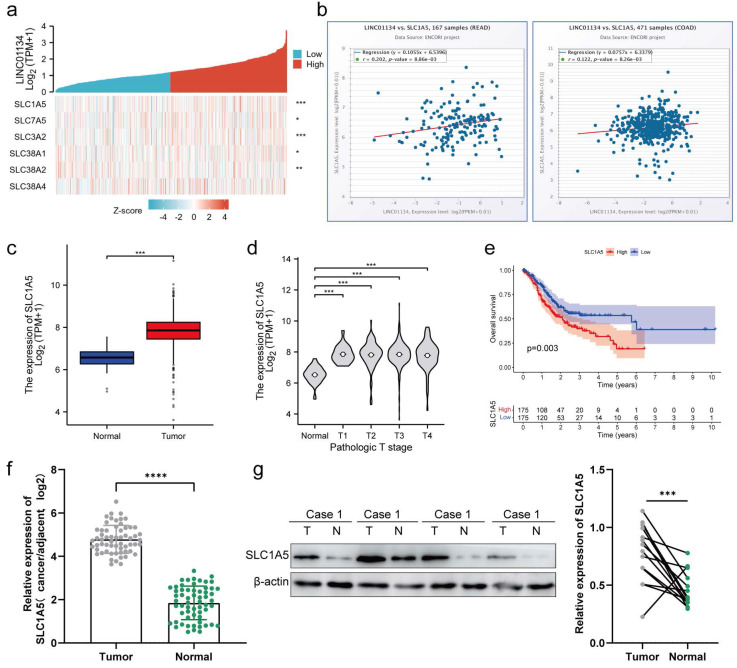
The expression of SLC1A5 is positively correlated with that of LINC01134 in CRC tissues and cell lines. (a) Co-expression analysis between LINC01134 and SLC family; RNA-RNA Co-Expression of LINC01134 and SLC1A5 both in COAD (b) and READ (c) was predicted based on starBase2.0 of ENCORI platform; (c-e) The expression levels and survival analysis of LINC01134 based on the Cancer Genome Atlas (TCGA) and the Genotype Tissue Expression (GTEx) portal via GEPIA and R software; The expression of SLC1A5 in CRC tissues (n=60) and compared healthy controls(n=60) was detected by qRT-PCR (f) and western blot (g). Each experiment was performed in triplicate. The data were shown as mean±SD. ***p<0.001; ****p<0.0001.

The Solute Carrier (SLC) family is comprised of high-affinity L-glutamine transporters that are overexpressed in various cancer tissues, regulating the proliferation and apoptosis of cancer cells^18^, ^19^. We hypothesized that LINC01134 impacted the proliferation and apoptosis of CRC cells through regulation of the SLC family. Co-expression analysis of LINC01134 and SLC families was conducted using the TCGA-COAD and TCGA-READ datasets, with the results indicating that the expression level of LINC01134 was most significantly positively correlated with SLC1A5 (alanine, serine, cysteine-preferring transporter 2[ASCT2]), as shown by the hot map from co-expression analysis (Figure 4a). RNA-RNA Co-Expression of LINC01134 and SLC1A5 in both COAD and READ on starBase2.0 of ENCORI platform were consistent with that based on TCGA-COAD and TCGA-READ datasets (Figure 4b). Similar to LINC01134, high levels of SLC1A5 indicated worse clinical stage (Figure 4c) and shorter overall survival (OS) (Figure 4e, P=0.003, log-rank test) in CRC patients. Furthermore, mRNA and protein expression levels of SLC1A5 were detected by qRT-PCR and western blot, and the results showed that SLC1A5 was upregulated in CRC tissue compared to adjacent normal colorectal tissues (Figure 4f, g). This indicates that LINC01134 may regulate the mRNA and protein expression of SLC1A5.

### LINC01134 enhances SLC1A5 mRNA stability via directly binding to the CDS of SLC1A5

**Figure 5:**
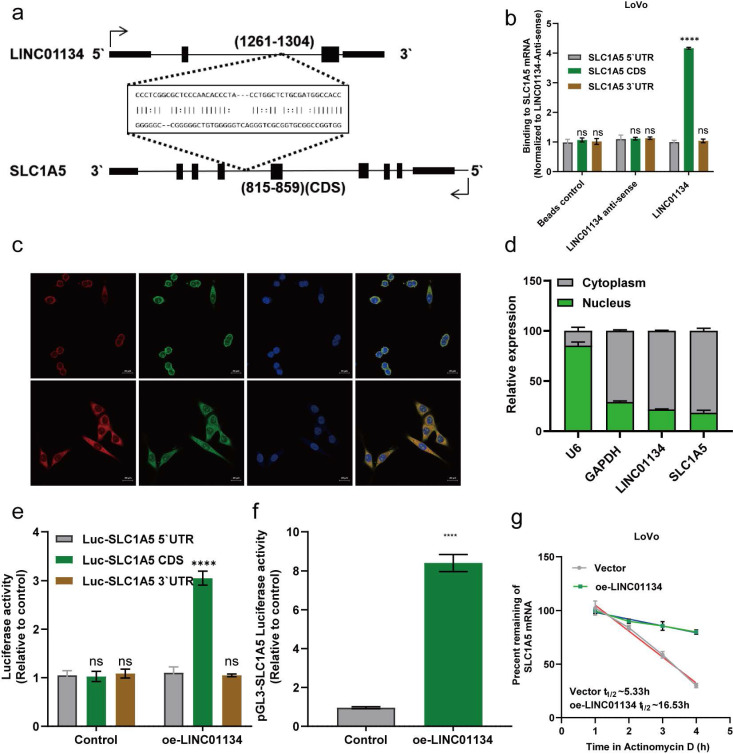
LINC01134 Enhances SLC1A5 mRNA stability via Directly Binding to the CDS of SLC1A5. (a) RNA-RNA interactions between LINC01134 and SLC1A5 were predicted based on RNA sequences via an online tool (http://rtools.cbrc.jp/); (b) The predicted interaction between LINC01135 and SLC1A5 mRNA was confirmed by vitro RNA-RNA interaction assay. Subcellular fractionation assay of LINC01134 and SLC1A5 was detected using fluorescence *in situ* hybridization (FISH) (c) and nuclear and cytoplasmic RNA isolation experiments (d); (e) The interaction between LINC01134 and SLC1A5 was further confirmed using luciferase reporter analysis; (f) The promoter activity of SLC1A5 was evaluated in CRC cells with LINC01134 overexpression or not; (g) SLC1A5 mRNA stability was assessed by the transcription inhibitor actinomycin (ActD). All experiments were conducted in triplicate. ns, not significant; ****p<0.0001.

We investigated the interaction between LINC01134 and SLC1A5. Based on previous findings that lncRNAs could target mRNAs through RNA-RNA interaction^20^, we hypothesized that LINC01134 directly binds to SLC1A5 mRNA and regulates its functions. Using an online tool (http://rtools.cbrc.jp/), we predicted a potential interaction between LINC01134 and the coding sequence (CDS) of SLC1A5 (Figure 5a). Subsequent subcellular fractionation assays using fluorescence *in situ* hybridization (FISH) and nuclear and cytoplasmic RNA isolation experiments showed that LINC01134 and SLC1A5 co-localized mostly in the cell cytoplasm, suggesting that LINC01134 might regulate post-transcription events (Figure 5c, d). *In vitro* RNA-RNA interaction assays confirmed that LINC01134 directly binds to the CDS of SLC1A5 (Figure 5b). Furthermore, luciferase reporter analysis revealed that the luciferase activity of the reporter containing SLC1A5 CDS was enhanced in LINC01134-overexpressing LoVo cells but not in those expressing the 5' UTR or 3'UTR (Figure 5e, f). Importantly, Actinomycin D was used to measure SLC1A5 mRNA stability, and the results showed that the decay rate of SLC1A5 mRNA decreased in LINC01134-overexpressing LoVo cells compared with those in the empty vector group (Figure 5g). Our findings indicate that LINC01134 directly binds to the 3'UTR of SLC1A5, enhancing its mRNA stability.

### Silencing SLC1A5 reverses the effect of LINC01134 in promoting CRC proliferation and inhibiting apoptosis

We conducted *in vitro* rescue experiments to investigate whether LINC01134 promotes proliferation and inhibits apoptosis through SLC1A5. Firstly, we transduced stably LINC01134-overexpressing LoVo cell lines with a lentivirus containing a LINC01134 overexpression construct or an empty vector control, which was then transfected with siRNA of SLC1A5 or siNC. qRT-PCR and Western blot were used to detect mRNA and protein expression levels of SLC1A5, respectively. The results showed that upregulated LINC01134 increased the mRNA expression levels of SLC1A5, which were mostly reversed by SLC1A5 silencing (Figure 5a). Secondly, CCK8, EdU, and colon formation assays revealed that the promotion of CRC cell proliferation was mostly reversed by SLC1A5 silencing (Figure 5b-d). Thirdly, flow cytometry analysis showed that the attenuation of cell apoptosis was reduced by SLC1A5 silencing (Figure 5e). Therefore, our findings confirm the hypothesis that LINC01134 promotes proliferation and inhibits apoptosis of CRC cells via activation of SLC1A5.

## Discussion

CRC is a prevalent gastrointestinal cancer that poses a significant threat to patients' quality of life and causes a considerable burden on society and healthcare systems. Although increasing studies have demonstrated that lncRNAs are critical drivers in the development and progression of CRC, yet their potential functional roles and molecular mechanisms in CRC remain unknown.

Previous studies revealed that LINC01134 was a significant oncogene in HCC, participating in tumor proliferation, migration, epithelial-mesenchymal transition (EMT), drug resistance. However, the roles of LINC01134 in CRC have not been systematically explored. In this study, we found that LINC01134 was significantly upregulated in CRC tissues compared with para-cancerous tissues. High expression of LINC01134 was associated with poor overall survival. Subsequent functional experiments showed that knockdown of LINC01134 inhibited the proliferation, migration, and EMT of CRC cells. Moreover, silencing LINC01134 suppressed subcutaneous tumor growth in nude mice.

Generally, lncRNAs exert their functions through three different mechanisms based on their subcellular distribution. Those primarily located in the nucleus regulate gene expression at the transcriptional level, while those primarily located in the cytoplasm regulate gene expression at the post-transcriptional level, including miRNA sponging and mRNA stability. In this study, we verified that LINC01134 was primarily distributed in the cytoplasm and promoted SLC1A5 expression by increasing its mRNA stability. Mechanistic experiments indicated that LINC01134 directly binds to SLC1A5 mRNA. Our findings have revealed a novel function of lncRNAs in modulating mRNA stability in CRC. This study offers new insights into how LINC01134 regulates its downstream targeted genes, which could provide novel biomarkers and targets for CRC diagnosis and treatment.

The SLC1A5 gene encodes a sodium-dependent neutral amino acid transporter that can act as a receptor for RD114/type D retrovirus^21^. Previous studies have shown that SLC1A5 is overexpressed in various malignant tumors, including gastric cancer^22^, esophageal cancer^23^, and head and neck squamous cell carcinoma(HNSCC)^24^. For example, Lu *et al.* found that SLC1A5 was significantly upregulated in gastric cancer tissues and correlated with malignant features such as deeper local invasion, higher lymph node metastasis, advanced TNM stages, and higher MCM-2 expression^22^. They also found that knockdown of SLC1A5 inhibited cell proliferation, invasion, and migration partly through the mTOR/p-70S6K1 signaling pathway^22^. Lin *et al.* revealed that SLC1A5 expression in cancerous tissues was significantly higher than in paired adjacent normal tissues and that silencing SLC1A5 suppressed esophageal cancer growth via cell cycle arrest and apoptosis^23^. Additionally, Zhang and colleagues demonstrated that SLC1A5 was overexpressed in HNSCC and associated with poor survival. They further identified that SLC1A5-dependent glutamine uptake and subsequent metabolism were essential for HNSCC tumorigenesis^24^. Consistent with these findings, increasing studies indicate that SLC1A5 plays a critical role in CRC. Witte *et al.* found that SLC1A5 was significantly overexpressed in CRC^25^. Furthermore, SLC1A5 knockdown inhibited CRC cell proliferation and migration and increased the efficacy of cetuximab on CRC^12^, ^26^. Therefore, given that LINC01134 functions through increasing SLC1A5 mRNA stability, targeting this pathway may be a promising therapeutic strategy for CRC.

In summary, our research has uncovered a new mechanism involving lncRNAs in CRC. We found that LINC01134 was significantly upregulated in CRC and that its inhibition could impede the progression of CRC both *in vitro* and *in vivo*, suggesting its potential therapeutic impact on CRC development. Mechanistically, LINC01134 promoted the upregulation of SLC1A5 by increasing its mRNA stability, thus regulating CRC progression. Overall, our findings demonstrate that LINC01134/SLC1A5 is involved in the progression of CRC, offering prospects for developing novel diagnostic and therapeutic targets for this disease.

### Limitations and future research

While our study has provided significant insights into the role of LINC01134 in CRC progression, several limitations must be acknowledged. First, our study primarily relied on *in vitro* and *in vivo* models, and further validation in clinical samples is necessary to confirm our findings. Second, the precise molecular mechanisms by which LINC01134 enhances the stability of SLC1A5 mRNA remain to be fully elucidated. Future research should focus on identifying the specific binding sites and interacting proteins involved in this regulation. Additionally, exploring the potential feedback loops and regulatory networks between LINC01134 and other key molecules in CRC could provide a more comprehensive understanding of its role in tumorigenesis. Finally, although our study suggests that targeting the LINC01134/SLC1A5 axis could be a therapeutic strategy for CRC, preclinical and clinical studies are required to assess the safety and efficacy of potential therapeutic interventions.

## Conclusion

Our study aimed to investigate the roles and mechanisms of CRC development. We observed that LINC01134 was highly expressed in CRC tissues and cell lines, and patients with high expression of LINC01134 had poor prognosis. Additionally, we found that LINC01134 could bind to SLC1A5 mRNA and enhance its stability. SLC1A5 is also highly expressed in CRC tissues and cell lines and is associated with poor prognosis. Our findings suggested that LINC01134 acts as an oncogene in CRC by directly binding to SLC1A5 mRNA and enhancing its stability. Therefore, LINC01134 might serve as a promising therapeutic target for CRC.

## Supplementary Material

Supplementary figures and tables.

## Figures and Tables

**Figure 6:**
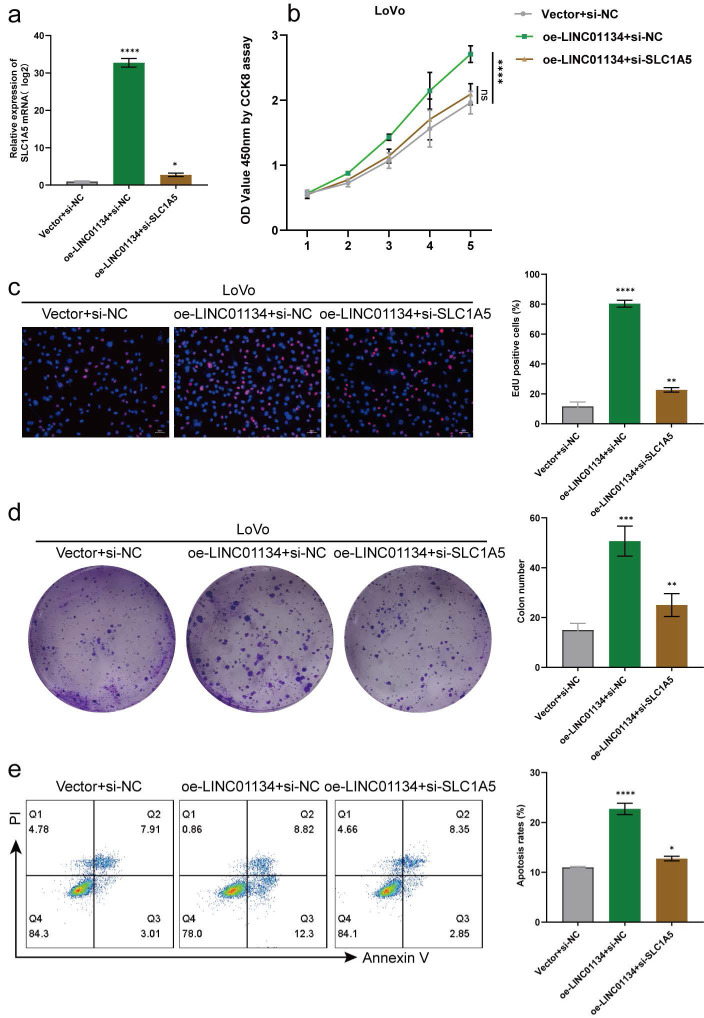
SLC1A5 Silencing Rescues the Roles of LINC01134 in Promoting CRC Proliferation and Inhibiting the Apoptosis. (a) The expression level of SLC1A5 was by qRT-PCR in stably LINC01134-overexpressed LoVo cell lines transfected with siRNA of SLC1A5 or siNC; The proliferation of CRC cell lines was measured by CCK8 assay (b), colon formation assay (c), and EdU staining (d); (e) The cell apoptosis of CRC cell lines was conducted by flow cytometry. All experiments were conducted in triplicate. ns, not significant; *p<0.05, **p<0.01, ***p<0.001, ****p<0.0001.

**Table 1:** Relationship between the LINC01134 levels and clinicopathological features of 60 CRC patients.

Variable	N	LINC01134 level		p value
		low	high	
All cases	60	30	30	
Age(years)				0.7892
≥65	22	12	10	
˂65	38	18	20	
Gender				0.7892
Male	38	18	20	
Female	22	12	10	
Tumor location				0.5921
Colon	22	12	10	
Rectum	38	18	20	
Tumor size (cm)				0.1954
≤5	32	19	13	
>5	28	11	17	
Pathological T category				0.0539
T1-T2	20	14	6	
T3-T4	40	16	24	
Lymph node metastasis				0.0379*
N0	29	19	10	
N1-2	31	11	20	
Distant metastasis				0.0575
M0	47	27	20	
M1	13	3	10	
Tumor stage				0.1188
I-II	27	17	10	
III-IV	33	13	20	
Histologic grade				0.3330
Well	19	13	6	
Moderate	34	14	20	
Poor	7	3	4	

*Indicated statistical significance (P < 0.05).
