# Supplementary material for: LINC01134 Directly Binds and Regulates SLC1A5 Stability to Promotes Colorectal Cancer Progression
Source: J Cancer. 2024 Oct 7;15(18):6135–47. doi: 10.7150/jca.100147 (PMC11493009; doi:10.7150/jca.100147)
Supplement: Supplementary file 1 — Supplementary figures and tables. [file jcav15p6135s1.pdf]

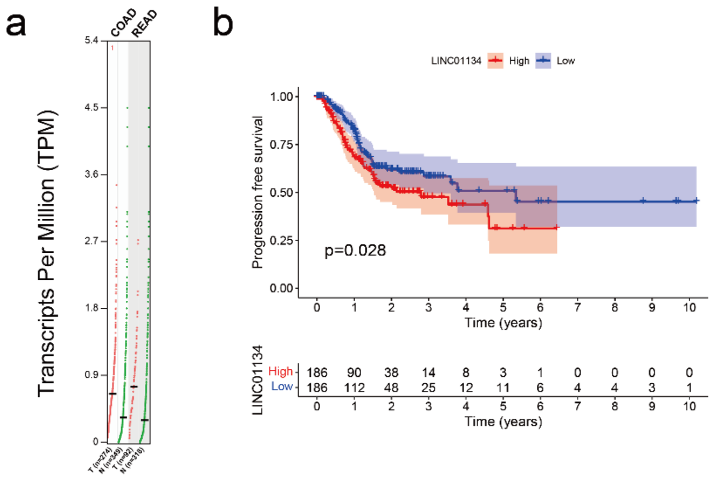

Supplementary Figure S1. (a) The expression profile and prognostic analysis of LINC01134 in colorectal cancer (CRC); (b) The survival analysis of LINC01134 in CRC patients.

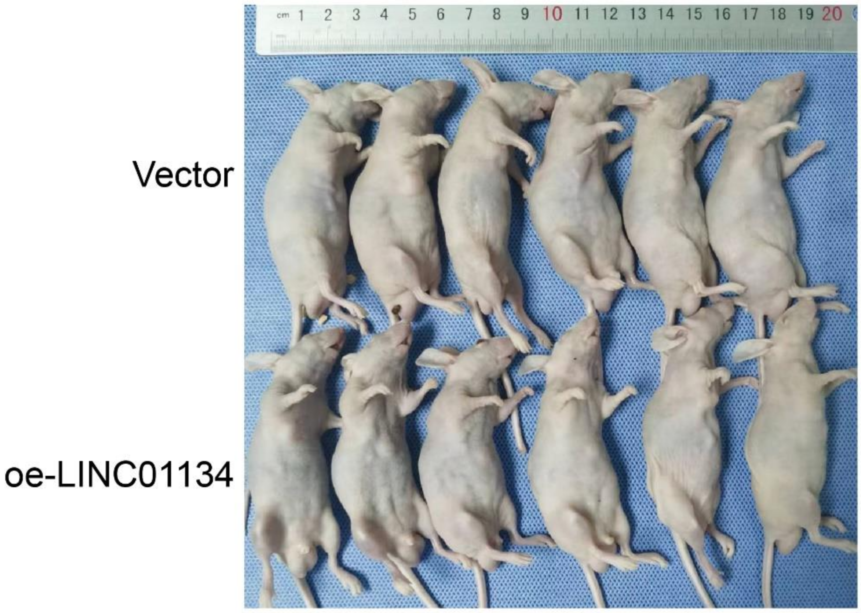

Supplementary Figure S2. Gross images of subcutaneous xenografted nude mouse models of human CRC.

Tables Legends

TABLE1. Relationship between the LINC01134 levels and clinicopathological features of 60 CRC patients.

Supplementary Table S1. Primer sequences for qRT-PCR

Primers	Sequence (5′-3′)
LINC01134	
Forwad	TTGGACCATGTCAGTGACGG
Reverse	CAGAGCCAGGTAGGGTGTTG
SLC1A5	
Forwad	GAGCTGCTTATCCGCTTCTTC
Reverse	GGGGCGTACCACATGATCC
GAPDH	
Forwad	GACAGTCAGCCGCATCTTCT
Reverse	GCGCCCAATACGACCAAATC
U6	
Forwad	TCCCTTCGGGGACATCCG
Reverse	AATTTTGGACCATTTCTCGATTTGT

Supplementary Table S2. List of sequences for shRNA and siRNA

Primers	Sequence (5′-3′)
sh-LINC01134-1	
sense	CACCGGACAGGTTTGAGCTAGAAACTTCAAGAGAGTTTCTAGCTCAAACCTGTCCTTTTTTGGA
anti-sense	TCCAAAAAAGGACAGGTTTGAGCTAGAAACTCTCTTGAAGTTTCTAGCTCAAACCTGTCC
sh-LINC01134-2	
sense	CACCGCGCATCCACTCATTCACTCATTCAAGAGATGAGTGAATGAGTGGATGCGCTTTTTTGGA
anti-sense	TCCAAAAAAGCGCATCCACTCATTCACTCATCTCTTGAATGAGTGAATGAGTGGATGCGC
sh-NC	
sense	CACCGTTCTCCGAACGTGTCACTTCAAGAGATTACGTGACACGTTCGGAGAATTTTTTGGA
anti-sense	TCCAAAAAATTCTCCGAACGTGTCACGTAATCTCTTGAAGTGACACGTTCGGAGAAC

Primers	Sequence (5′-3′)
si-SLC1A5	
Forward	GCACAGAGCCUGAGUUGAUTT
Reverse	AUCAACUCAGGCUCUGUGCTT
si-NC	
Forward	CGCACTGTGCAAGCCTCTA
Reverse	ACGUGACACGUUCGGAGAT

Supplementary Table S3. Antibodies used in this study.

Antigens	Manufacturers	Application
GAPDH	#8457S, Cell Signaling Technology, Beverly, MA, USA	1:1000 for WB
anti-rabbit IgG HRP conjugated	#7074, Cell Signaling Technology, Beverly, MA, USA	1:1000–1:3000 for WB
SLC1A5	#8057, Cell Signaling Technology, Beverly, MA, USA	1:1000 for WB
BrdU	ab2284, ABCAM, Cambridge, UK	5 µg per test
Ki-67	#9449S,, Cell Signaling Technology, Beverly, MA, USA	1:800-1:3200 for IHC

WB: Western Blot; IHC: Immunohistochemistry.
